# Impact of coronavirus disease 2019 on the utilization of hospital services and development of optimal pandemic control strategy in Chinese tertiary hospitals during the Omicron wave

**DOI:** 10.1186/s12913-024-11289-1

**Published:** 2024-07-23

**Authors:** Dan Yu, Dong Wang, Yi Chen, Ting Dong, Yinchu Zhang, Mengjun Huang, Anhua Wu, Yi Ouyang, Wendong Chen, Zhaoxin Qian

**Affiliations:** 1 Xiangya Hospital of Central South University, Changsha, China; 2 Changsha Normin Health Technology Ltd, Changsha, China; 3 The People’s Hospital of Liuyang, Changsha, China; 4 Changsha Central Hospital, Changsha, China

**Keywords:** Coronavirus disease 2019, COVID-19, Pandemic control, Medical service capacity, Revenue, Tertiary hospital

## Abstract

**Background:**

This study aimed to assess the impact of coronavirus disease 2019 (COVID-19) on hospital service utilization and revenue in Chinese tertiary hospitals and develop an optimal pandemic control strategy (OPCS) for the peak period of the Omicron wave.

**Methods:**

Retrospective data from three Chinese tertiary hospitals (provincial, city, and county level) were analyzed for three phases: pre-outbreak (Jan-Apr 2019), outbreak (Jan-Apr 2020), and post-outbreak (Jan-Apr 2021). OPCS was developed under the guidance of the China government pandemic control policy during post-break phase of COVID-19. A decision-tree model was constructed to compare OPCS to strict pandemic control strategy during outbreak phase for the hospital service utilization and hospital revenue in a provincial tertiary hospital during the Omicron wave.

**Results:**

Outpatient, emergency room (ER) visits, hospitalizations, and intensive care admissions dropped by 33.8–53.4% during the outbreak, with the provincial hospital being the most affected. Hospital revenue also declined, especially for the provincial hospital (40.1%). Post-outbreak, most services recovered, but ER visits remained lower (11.6% decrease for provincial hospital, 46.5% for county hospital). Total income and expenditure decreased, with the provincial hospital experiencing the most significant revenue reduction (45.7%). OPCS showed greater utilization of medical services (31.6 times more outpatient visits; 1.7 times more inpatient days; 3.4% more surgery volume) and higher revenue (¥220.8 million more) compared to the strict pandemic control strategy.

**Conclusions:**

COVID-19 measures were associated with less hospital service utilization and revenue in Chinese tertiary hospitals. The developed OPCS in Chinese tertiary hospitals, focusing on isolating infected inpatients but not shutting down the hospital facilities exposed to virus, could be effective in optimizing hospital service utilization and hospital revenue during the Omicron wave.

## Background

China experienced a major outbreak of coronavirus disease 2019 (COVID-19), with the initial cases reported in the city of Wuhan, Hubei province, in December 2019 [1]. The virus quickly spread within Wuhan and to other parts of China. The Chinese government took strict measures to contain the virus, including the lockdown of Wuhan, travel restrictions, mass testing, contact tracing, and quarantine measures. In the meantime, local governments in other cities across China developed pandemic control measures under the guidance of the Chinese central government to prevent the spread of the virus across the country. Over time, China managed to significantly bring down the number of new cases, and by mid-2020, the situation within the country had largely stabilized [2]. The government gradually eased restrictions and reopened businesses, schools, and public spaces. However, sporadic outbreaks and localized clusters continued to occur, prompting swift local responses to contain the virus.

Strict pandemic control measures were implemented in tertiary care hospitals across China following the initial outbreak of COVID-19 [3]. These measures aimed to protect healthcare workers, prevent healthcare-associated infections, limit nosocomial transmission, preserve healthcare capacity, and maintain public trust. While these measures were effective in containing the virus, they also restricted the utilization of hospital services, which is closely linked to hospital revenue. As most tertiary care hospitals in China are publicly funded and primarily operate on a pay-for-service model, the strict pandemic control measures led to financial distress when government subsidies were lacking or insufficient. Tertiary care hospitals had to delicately adjust their pandemic control measures as the situation evolved and find a balance between the requirements of pandemic control and maintaining medical services capacity following the COVID-19 outbreak. Therefore, assessing the impact of the COVID-19 outbreak on hospital services capacity and revenue can help tertiary care hospitals prepare for future pandemics similar to COVID-19. In addition, the population immunity against COVID-19 in China has been established after several waves of infections and universal vaccination. The pandemic control strategies should be adjusted accordingly to recover the medical services capacity of tertiary hospitals and address the medical needs of the Chinese patients.

## Methods

This study consisted of two parts. One part was a retrospective analysis assessing the impact of the COVID-19 outbreak on the utilization of hospital services and financial metrics in the selected three tertiary hospitals in Hunan province, China. The second part assessed the impact of the developed optimal pandemic control strategy (OPCS) on the utilization of hospital services and hospital revenue in a provincial tertiary hospital during the Omicron wave that started in the winter of 2022. The retrospective analysis focused on three different levels of tertiary hospitals (provincial, city, and county level), and defined three distinct time periods [pre-outbreak phase (January 1 to April 30, 2019) served as a baseline, representing the time before the COVID-19 outbreak; outbreak phase (January 1 to April 30, 2020) corresponded to the time during the peak of the COVID-19 outbreak when medical services were likely affected by the surge in cases; post-outbreak phase (January 1 to April 30, 2021) represented a later period after the initial outbreak, which could reflect any lingering effects on medical services and financial metrics] for data collection. The evaluation of OPCS was assessed using a decision-tree model based on the real-world utilization of medical services and financial metrics in the provincial-level tertiary hospital included in the retrospective analysis. It was not appropriate or possible to involve patients or the public in the design, or conduct, or reporting, or dissemination plans of our research. However, this study was approved by the ethics review committee of Xiangya Hospital, Central South University (Application number: 202,008,459). The informed consent to participate was waived by the ethics review committee of Xiangya Hospital because of the retrospective nature of this study.

### Data sources

This study assessed the utilization of medical services using the administrative databases of the three selected hospitals which represented the three ranks of tertiary hospitals (provincial level, city level, and county level) in Chinese public healthcare system regarding the size, service capacity, and geographic characteristics. Another reason for selecting these three hospitals was that the established collaborations in the three hospitals allowed this study to access their administration and financial databases for medical services utilization and hospital operation. The key metrics analyzed included outpatient visits (the number of patients visiting the hospital for non-admission services), emergency room (ER) visits (the number of patients seeking immediate medical attention at the ER), hospital admissions (the number of patients admitted to the hospital for treatment and care), and surgery volume (the number of surgeries performed during each phase). Additionally, this study extracted financial metrics using the financial statements of the studied hospitals during the defined three phases. The collected key financial metrics included hospital operation costs (the expenses incurred by the hospital to maintain its services and operations), hospital revenue (the income generated by the hospital through the provision of medical services), government assistance funds (financial aid received from the government to support hospital operations), and costs of pandemic control measures (the expenses related to implementing measures to control the spread of COVID-19 within the hospitals). These data sources were used to conduct the planned retrospective analysis on the utilization of hospital services and financial metrics during the defined three phases of COVID-19 and develop the decision analytic model assessing OPCS in Chinese provincial hospital during Omicron wave.

### Developing hospital optimal pandemic control strategy

The Chinese tertiary hospitals employed strict pandemic control measures during the initial COVID-19 outbreak by reducing medical services, conducting strict polymerase chain reaction screening for COVID-19 among visiting patients, and quarantining hospital settings and hospital staff exposed to confirmed COVID-19 cases. With the control of COVID-19 achieved through increased intensive care capacity and widespread COVID-19 vaccination, the central government of China released optimal pandemic control guidance in November 2022 [4]. Thus, this study organized an expert committee from three tertiary hospitals to develop a hospital OPCS based on the government’s guidance. Different from strict pandemic control measures, the developed OPCS didn’t quadrant hospital facilities and hospital staff exposed to COVID-19 but set up a separate transitional ward to manage infected patients and hospital staff until they fully recovered with negative COVID-19 test results.

### Developing decision analytic model to assess OPCS in provincial hospital

This study constructed a decision analytic model to compare the developed OPCS with the implemented strict pandemic control strategy during the outbreak of COVID-19 (strict control strategy) for the utilization of hospital medical services and hospital revenue during the 3-month peak time of Omicron wave, which spread across China following the release of the government’s optimal pandemic control guidance in the winter of 2022 [5]. Under OPCS, the hospital would set up a separate transitional ward to manage COVID-19 infections for infected patients and hospital staff until they fully recovered from the infection with poof of negative COVID-19 test results. There would be no separate isolation of any hospital facilities or medical staff exposed to virus for OPCS. While under the strict control strategy, any hospital facilities with exposure to confirmed positive COVID-19 patients, including outpatient clinics, inpatient wards, and operating rooms, would be shut down for five days, and doctors who had contact with positive patients would be isolated for 8 days. This decision analytic model used a decision-tree model design to simulate the utilization of medical services and financial metrics in a provincial tertiary hospital under the two pandemic control strategies. The reported incidence rates of COVID-19 in the general population (884 per 100,000 persons) and hospital staff (0.44%) during the peak time of the fifth wave COVID-19 in Hong Kong [6, 7] were used to estimate the daily incidence of COVID-19 in the model according to the daily volume of visiting patients and number of hospital staff during Omicron wave. the model simulation time horizon was set to three months to align with the peak time of Omicron wave in China. The reduced hospital services under OPCS were estimated through the occupied hospital facilities by infected patients and hospital staff. The duration of isolated hospital facilities and hospital staff and their daily services volumes without isolation were used to estimate the reduction of hospital services due to the exposure to COVID-19 under the strict control strategy. The unit revenue and costs associated with each type of hospital service were estimated according to real-world utilization of medical services and financial statements of the studied provincial tertiary hospital (Xiangya Hospital) during the late phase of COVID-19 in the retrospective analysis.

### Data analysis

This study utilized descriptive statistical methods to analyze the medical service capacity and financial metrics of the three selected hospitals. The assessment involved comparing changes in the utilization of medical services and financial metrics during different phases of the COVID-19 pandemic: the pre-outbreak phase, the outbreak phase, and the post-outbreak phase. This study employed statistical software R (version 4.1.0) [8] to conduct the descriptive statistical analyses, allowing them to explore and summarize the data, providing valuable insights into the impact of the pandemic on medical service utilization and hospital finances.

In addition to the descriptive statistical analysis, the study used the constructed decision analytic model to perform a base-case analysis, providing point estimations for the differences in medical service utilization and hospital revenue between two pandemic control strategies: OPCS and the strict control strategy. By employing this model, this study could better understand the potential consequences of each control strategy and identify which one might be more effective for managing the pandemic’s impact on hospital services and finances. Furthermore, the study conducted a one-way sensitivity analysis to assess the robustness of the base-case analysis. In this analysis, each model input was varied within a defined range of +/-25% of its mean value. This sensitivity analysis allowed this study to test the model’s results under different scenarios, accounting for potential uncertainties or fluctuations in the data, providing a more comprehensive understanding of the potential outcomes of the pandemic control strategies, taking into consideration possible variations in the input parameters. The decision analytical model was constructed using Microsoft Excel.

#### Results

The results of the retrospective analysis on the utilization of medical services and financial statements in the defined three phases were reported by the changes from pre-outbreak. phase The impact of OPCS on the utilization of medical services and hospital revenue was reported through the comparisons with strict control strategy in the base case analysis and one-way sensitivity analysis of the constructed decision analytic model.

### Changes of the utilization of hospital services after the outbreak of COVID-19

Compared to the pre-outbreak phase, the outbreak phase of COVID-19 was associated with a substantial reduction in the utilization of medical services in the selected three-level tertiary hospitals. During the outbreak phase, outpatient visits were reduced by 26.4–53.4%; ER visits were reduced by 33.8–50.3%; hospitalizations were reduced by 22.8–43.0%; surgery volume was reduced by 24.3–45.7%; and intensive care unit (ICU) admissions were reduced by 43.7% for the provincial tertiary hospital and 20.2% for the city tertiary hospital. Overall, the utilization of medical services (except ER visits) in the selected provincial tertiary hospital was reduced the most. During the defined late phase of COVID-19, outpatient visits (provincial tertiary hospital: 9.6%, city tertiary hospital: 6.2%, county tertiary hospital: 4.3%)) and surgery volume (provincial tertiary hospital: 18.6%, city tertiary hospital: 9.3%, county tertiary hospital: 10.2%) in the three-level tertiary hospitals were fully recovered with a moderate increase. However, the ER visits in the provincial tertiary hospital (-11.6%) and county hospital (-46.5%) were still lower than the ER visits during the pre-outbreak phase. The utilization of hospitalization (-10.2%) and ICU (-5.4%) in the city tertiary hospital was not recovered to the level before the outbreak. The utilizations of medical services in the three selected tertiary hospitals in the defined three study phases are summarized in Table 1.

**Table 1:** Changes of hospital service utilization before and after COVID-19 outbreak

Medical service	Outbreak phase vs. Pre-outbreak phase			Post-outbreak phase vs. Pre-outbreak phase		
	Provincial tertiary hospital	City tertiary hospital	County tertiary hospital	Provincial tertiary hospital	City tertiary hospital	County tertiary hospital
Outpatient visits	-53.4%	-37.8%	-26.4%	9.6%	6.2%	4.3%
Emergency room visits	-33.8	-50.1%	-50.3%	-11.6%	8.0	-46.5%
Hospitalizations	-43.0%	-26.4%	-22.8%	8.7	-10.2%	15.0%
Surgery volume	-45.7%	-24.3%	-31.5%	18.6%	9.3%	10.2%
Intensive care utilization	-43.7%	-20.2%	Not applicable	0.6	-5.4%	Not applicable

### Changes of hospital revenue after the outbreak of COVID-19

Compared to the financial statements during the pre-outbreak phase, the revenues of the three selected tertiary hospitals were reduced by 17.9–40.1% during the outbreak phase. Even with substantial increases in basic government financial subsidies (97.3%) and project-based government subsidies (214.5%), the selected provincial tertiary hospital was associated with the most revenue reduction (40.1%) among the three-level tertiary hospitals, mainly due to the reduced service incomes from outpatient care (45.7%) and inpatient care (46.2%). However, the revenues of the three selected hospitals in the post-outbreak phase increased by 4.8–15.0%, mainly due to the increased service incomes from outpatient care and inpatient care. The county tertiary hospital had the largest increase in hospital revenue among the three selected hospitals. The three selected hospitals had comparable changes in total expenditure during the outbreak phase (reduced by 14.8–15.4%) and post-outbreak phase (increased by 1.1–3.1%). However, the distribution of expenditures in the three hospitals was slightly different. The provincial tertiary hospital was associated with a substantially increased employee salary expenditure (34.2%) during the outbreak phase, while the other two selected hospitals had slightly reduced expenditure on employee salary (city tertiary hospital: 8.5%; county tertiary hospital: 1.5%). The financial statements of the three selected tertiary hospitals during the defined phases are summarized in Table 2.

**Table 2:** Changes of hospital financial metrics before and after COVID-19 outbreak

Financial metrics	Outbreak phase vs. Pre-outbreak phase			Post-outbreak phase vs. Pre-outbreak phase		
	Provincial tertiary hospital	City tertiary hospital	County tertiary hospital	Provincial tertiary hospital	City tertiary hospital	County tertiary hospital
Total revenue	-40.1%	-17.9%	-24.8%	4.8%	7.3%	15.0%
Outpatient service income	-46.2%	-30.6%	-22.7%	4.7%	3.5%	11.9%
Inpatient service income	-45.5%	-17.1%	-25.1%	3.7%	4.4%	20.2%
Basic government financial subsidy	97.3	-25.0%		37.2%	1.4%	
Project-based government subsidy	214.5%	63.4%		5.8%	259.3%	
Total expenditure	-15.4%	-14.8%	-14.8%	1.1%	3.1%	3.1%
Employee salary	34.2	-8.5%	-1.5%	3.3%	12.4%	34.6%
Drug costs	-47.6%	-24.6%	-27.5%	-11.9%	-9.1%	0.6%
Medical supply costs	-39.1%	-23.0%	-21.9%	27.0%	7.6%	19.6%

### Impact of hospital pandemic control strategies on hospital services utilization and hospital revenue

Based on the incidence rate of the fifth wave of COVID-19 during the peak period, the model predicted that the provincial tertiary hospital would have 18,904 infected patients (13,543 outpatients and 5,361 inpatients) and 1,331 infected hospital staff over a three-month simulation time. The base case analysis estimated that OPCS would provide more medical services compared to the strict control strategy. Specifically, OPCS is projected to have higher outpatient visits (853,652 vs. 26,150), inpatient days (237,096 vs. 86,758), and surgery volume (29,765 vs. 28,781) per quarter. Moreover, OPCS is expected to gain more revenue (¥49.309 million) per quarter than the strict control strategy, which is projected to incur a loss of -¥171.466 million per quarter. The detailed results of the base case analysis are summarized in Table 3.

**Table 3:** Base case analysis results for the two pandemic control strategies

Pandemic control strategy	OPCS	Strict control strategy	Difference
Total number of infected patients	18,904	18,904	0
Number of infected outpatients	13,543	13,543	0
Number of infected inpatients	5361	5361	0
Hospital services (× 1,000)			
Outpatient visits	853.652	26.15	827.502
Inpatient care days	237.096	86.758	150.338
Surgery volume	29.765	28.781	0.984
Reduced health services due to infected physician (× 1,000)			
Outpatient visits	103.822	931.324	-827.502
Inpatient care days	25.635	201.451	-175.816
Surgery volume	0.345	1.329	-0.984
Hospital revenue (million)			
Outpatient care	¥524.05	¥16.05	¥508.00
Inpatient care	¥828.68	¥303.23	¥525.45
Surgery	¥222.20	¥214.85	¥7.35
Infected inpatient care	¥43.53	¥0.00	¥43.53
Government financial subsidies	¥19.45	¥19.45	¥0.00
Total revenue	¥1,637.92	¥553.59	¥1,084.33
Hospital expenditure (million)			
Outpatient care	¥422.67	¥12.95	¥409.73
Inpatient care	¥668.36	¥244.57	¥423.80
Surgery	¥102.89	¥99.49	¥3.40
Infected inpatient care	¥35.11	¥0.00	¥35.11
Pandemic control	¥1.60	¥10.08	-¥8.48
Fixed operation costs	¥357.98	¥357.98	¥0.00
Total expenditure	¥1,588.61	¥725.06	¥863.56
Financial balance (millions)	¥49.31	-¥171.47	¥220.78

**Fig. 1:**
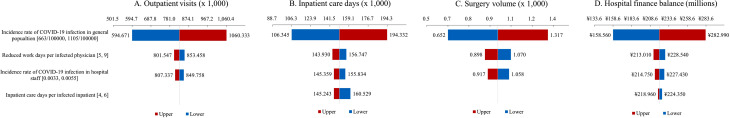
One-way sensitivity analysis for differences in hospital services and revenue between OPCS and strict control strategies

One-way sensitivity analysis identified that the most influential model input driving the model outputs is the incidence rate of COVID-19 in the general population. However, this factor does not alter the trend that OPCS can provide more health services and generate more revenue than the strict pandemic control strategy in the provincial tertiary hospital during Omicron wave. The other model inputs with a moderate impact on the model outputs included reduced workdays of the infected physician, incidence rate of COVID**-19** in hospital staff, and length of stay in transitional ward for infected patients. The results of the one-way sensitivity analyses for model outputs are illustrated in Fig. 1.

## Discussion

During the initial COVID-19 outbreak, tertiary hospitals across China implemented strict control measures to limit virus transmission, protect vulnerable patients, maintain healthcare services, and prevent overwhelming the healthcare system [9]. As a result, hospital services were substantially under used and hospitals operated at a loss revenue even with government financial support, regardless of hospital rank. With the stabilization of COVID-19 in China, the tertiary hospitals were able to fully recover core hospital services and achieve financial balance even under relatively strict pandemic control measures. Based on the model simulations for OPCS with only quarantining infected inpatients and hospital staff and previously used strict control strategy during pandemic outbreak, OPCS was expected to be a better strategy by optimizing the utilization of hospital services and hospital revenue during Omicron wave across China.

Chinese authorities implemented a series of pandemic control measures that directly affected the utilization of hospital services during the outbreak of COVID-19. For example, strict lockdowns and quarantine measures restricted movement, preventing people from leaving or entering areas with high COVID-19 case numbers. Travel restrictions suspended public transportation services, closed transportation hubs, and limited intercity travel. Social distancing measures reduced gatherings in public spaces and workplaces. These control measures significantly limited patient access to healthcare facilities and reduced the service capacity in Chinese tertiary hospitals [9, 10]. Additionally, the fear of contracting COVID-19 in healthcare settings may have led some patients to avoid seeking medical care altogether, even for urgent or chronic health conditions [11]. Tertiary hospitals had to adapt rapidly to respond to the pandemic, resulting in some routine medical services and specialties being temporarily redirected to manage COVID-19 cases or support the fight against the outbreak in other areas. As a result, this study observed a substantial reduction in hospital service utilization and hospital revenue in the three selected tertiary hospitals, irrespective of their ranks. Given that nearly half of patients visiting top-ranked tertiary hospitals, such as provincial tertiary hospitals, come from remote areas [12], the utilization of hospital services and hospital revenues in these hospitals could have been most affected by the travel restrictions during the pandemic outbreak. Additionally, provincial tertiary hospitals had to allocate more resources to implement pandemic control measures for large patient volume and support pandemic control in other cities upon the request from government. Thus, achieving financial balance would be more challenging for these top-ranked tertiary hospitals during the outbreak of COVID-19, even with government financial support.

This study observed that outpatient care and surgery volume in the selected three tertiary hospitals were fully recovered, with a moderate increase during the post-outbreak phase, likely due to the well-controlled pandemic in Hunan province, where the selected hospitals are located. Since it was unlikely for hospitals to full resume the routine medical services, the moderate increase of outpatient care and surgery volume might be driven by the patients who had to delay their visits to hospitals during the pandemic outbreak. However, the utilization of hospitalization and intensive care in the city tertiary hospital did not fully recover to pre-outbreak levels. This might be explained by the differences in patient preference to the hospital level. Chinese patients with severe diseases tend to prefer visiting top-ranked tertiary hospitals [13]. The urgency of medical needs in these patients could drive back the utilization of hospitalization and intensive care in provincial hospitals, but not in city hospitals, when the pandemic was stabilized.

Before Omicron wave started in China, the China government started to ease the restrictions by releasing the optimal pandemic control guidance in November 2022 [4] after achieving high vaccination rates in healthcare workers and vulnerable populations through national COVID-19 vaccination campaigns [14–16]. Thus, many tertiary hospitals, including the three selected hospitals, developed OPCS by only isolating the infected inpatients and hospital staff without interrupting the hospital routine services for other uninfected patients. Based on the simulation of the constructed decision analytic model in this study, OPCS could increase outpatient care capacity by over 30 times to meet the patients’ medical needs during Omicron wave. The generated revenue from these increased hospital services could provide fundamental support for sustainable hospital operation. When the Omicron wave started in China, most of Chinese tertiary hospitals had to use loose control strategies to handle the surge of Omicron cases and medical needs of non-infected patients. Luckily, the Chinese tertiary hospitals successfully passed the Omicron wave without causing substantial loss of patient lives [17] and financial crisis for hospital operation. Because the capacity of hospital services was highly correlated to the hospital facilities and hospital staff, the isolated hospital facilities and hospital staff due to virus exposure under the strict control strategies could have direct and strong impact on hospital capacity and finance. This well aligned with the strongest impact of the incidence rate of COVID-19 in our sensitivity analysis. This also aligned with the moderate impact of the isolation time of hospital facilities and hospital staff under the strict control strategies in the sensitivity analysis. However, these findings might not be used to guide the improvement of OPCS, which wouldn’t isolate the hospital facilities and hospital staff that were exposed to infected patients.

Even though the results in our study were consistent with the expected impact of the pandemic on the service capacity of hospitals and the potential financial benefits of OPCS, this study still has the following major limitations that should be considered for interpretation and dissemination. First, our study selected only three hospitals in one province to represent the hospital ranks in the Chinese public healthcare system, which consists of thousands of hospitals in over 30 provinces. Thus, the study results might have limited generalizability due to the small number of selected hospitals, different epidemiology of COVID-19, and varied pandemic control strategies. Second, the constructed decision-analytic model assessing OPCS didn’t take into account health benefits that should be an important consideration for pandemic control. Thus, the constructed OPCS model is inappropriate for guiding the development of pandemic control policies, which focus on balancing health benefits, patient medical needs, and health resources utilization. Third, the constructed OPCS model had limited flexibility as it only integrated one strategy to treat infected patients and hospital staff in isolated areas for OPCS. Actual pandemic control strategies should consider more options to address government pandemic control policies. Finally, the constructed OPCS model was not validated using real-world hospital services and financial data during the Omicron wave. Future studies could further improve our OPCS model by addressing the limitations listed above, making it more practical and useful from various perspectives to support hospital management in future pandemics.

## Conclusions

In conclusion, this study provides valuable insights into the effects of the COVID-19 pandemic on medical service utilization and hospital revenue in Chinese tertiary hospitals. The results indicate that the pandemic significantly disrupted medical service utilization and hospital revenue during the outbreak phase, with varied recovery rates across different services and hospitals in the post-outbreak phase. Additionally, the decision analytic model highlights that OPCS may lead to increased medical service utilization and hospital revenue compared to the strict control strategy during the peak period of the Omicron wave. Thus, OPCS without isolating hospital facilities and staff should be considered to maximize routine medical services to meet the medical needs of patients and provide sufficient revenue to support hospital operations in future COVID-19 waves.

## Data Availability

The data and materials supporting the findings of this study are available upon reasonable request. Requests for data and materials should be addressed to the corresponding author of this manuscript.

## Abbreviations

COVID-19: Coronavirus Disease 2019
ER: Emergency Room
ICU: Intensive Care Unit
